# Oyster Mushroom (*Pleurotus ostreatus*) and Okara Flour as Nutritional Enhancers in Wheat Biscuits: A Study on Storage Stability

**DOI:** 10.3390/foods14030539

**Published:** 2025-02-06

**Authors:** Emmanuella Ifunanya Nwaudah, Ifeoma Elizabeth Mbaeyi-Nwaoha, Deborah Chinwendu Ofoegbu, Helen Onyeaka

**Affiliations:** 1 Department of Food Science and Technology, Faculty of Agriculture, University of Nigeria, Nsukka 410105, Enugu State, Nigeria; emmanuelanwaudah@gmail.com (E.I.N.); ifeoma.mbaeyi-nwaoha@unn.edu.ng (I.E.M.-N.); deborahofoegbu4@gmail.com (D.C.O.); 2 School of Chemical Engineering, University of Birmingham, Birmingham B152 TT, UK

**Keywords:** formulated, enrichment, nutrient-contained, ambient temperature, malnutrition epidemic

## Abstract

In response to the scarcity and high cost of wheat in Nigeria, this study investigates the potential of oyster mushroom (*Pleurotus ostreatus*) and okara flour to enhance the nutritional quality and storage stability of wheat biscuits. By incorporating 10–50% oyster mushroom powder into wheat flour, this study observed significant increases in the nutritional profile of the biscuits. The protein content notably increased from 8.26% to 16.12%, while the crude fibre and ash content also saw over a 50% increment. Storage studies revealed that biscuits (baked for 18 min at 180 °C) packaged in cartons within polyethene were more shelf-stable than those in low-density polyethylene (LDPE) bags, maintaining quality over two months at ambient temperature. The inclusion of oyster mushroom and okara flour in wheat biscuits significantly enhances their nutritional value and shelf life, presenting a viable solution to the challenges of wheat scarcity and global malnutrition. The optimal mushroom flour enrichment level was identified at 20% to maintain consumer appeal.

## 1. Introduction

In Nigeria, the reliance on wheat as the primary raw material in the pastry and bakery industries faces significant challenges. Wheat cultivation is minimal, and its importation is costly [1], leading to a pressing need to explore alternative local sources of baking flour. The high cost and scarcity of wheat in Nigeria pose immense challenges to the baking industry, and the search for alternative basic ingredients has thus raised much attention. Wheat flour is expensive due to import duties, transportation costs, and currency exchange rates, while okara and oyster mushrooms are affordable and widely available [1]. This study aims not only to address this gap but also to enhance the nutritional quality and storage stability of wheat biscuits. A promising solution lies in using underexploited local ingredients such as okara, mushroom, and acha. Okara, the insoluble by-product of soymilk production, is rich in dietary fibre (50%), protein (25%), and fat (10%), along with essential minerals [2]. Despite its nutritional potential, okara’s application in food products remains limited, and its disposal poses environmental concerns [3,4]. Researchers like Sengupta et al. [5] have highlighted okara’s suitability as an inexpensive, nutrient-rich supplement in various food products. Moreover, the inherent limitations of wheat flour, particularly its deficiency in essential amino acids like lysine and tryptophan, make the prospect of composite flours appealing [6]. In this regard, mushrooms, especially the widely cultivated oyster mushroom (*Pleurotus ostreatus*), offer a rich profile of high-quality protein, dietary fibre, vitamins, and minerals [7,8]. Oyster mushrooms are particularly esteemed in Nigeria, thriving in both temperate and tropical climates and often used as a nutritious meat substitute in local cuisine while offering a sustainable alternative for food formulations [9,10].

This study, therefore, focuses on creating a novel blend of wheat, oyster mushroom, and okara flour for biscuit production. It aims to leverage the unique properties of these ingredients to improve the nutritional profile and storage stability of biscuits, providing a viable alternative to traditional wheat-based biscuits and contributing to the broader utilization of okara and mushrooms in the Nigerian food industry.

## 2. Materials and Methods

### 2.1. Procurement of Raw Materials

Wheat flour and soybean seeds were bought from Ogige market in Nsukka town, Enugu State, Nigeria. The mushrooms were obtained from Enugu City, Enugu State, Nigeria. The quality standards and specifications of the raw materials (wheat flour, okara flour, and oyster mushrooms) were meticulously considered to ensure the accuracy and comparability of the experimental results.

### 2.2. Preparation of Wheat Flour

The commercial wheat flour was sieved through a 60-mesh sieve (British standard sieve).

### 2.3. Production of Flour from Mushroom

The mushroom powder was prepared following the procedure outlined by Okeke [9]. Initially, the dried mushrooms were ground using a hammer mill, and, subsequently, the resulting powder was passed through a 60-mesh sieve (British Standard Screen). The powder was then airtight-sealed in a low-density polyethylene bag and stored in a refrigerator at 4 °C until it was ready for use.

### 2.4. Production of Okara from Soybean Seed

Okara was prepared using a modified method based on Uchendu [11]. Initially, five kilograms (5 kg) of soybeans were cleaned, soaked for 18 h, and subsequently dehulled. The dehulled beans were then milled and passed through a 60-mesh sieve (British Standard Sieve). The remaining residue was collected and subjected to drying in an oven at 105 °C for 3 h. Once dried, the residue, now referred to as okara, was allowed to cool and then sealed in an airtight container for storage.

### 2.5. Formulation of Composite Flour

Wheat and okara composite flours were formulated to contain varying weights of wheat and okara. Samples of 90:10, 80:20, 70:30, 60:40, and 50:50 *w*/*w* wheat to okara flour, respectively, and 100% wheat flour served as control.

### 2.6. Blending Composite Flour and Mushroom

The most acceptable ratio of wheat/okara flour from 50 to 100% was blended with graded levels of 0, 10, 20, 30, 40, and 50% oyster mushroom flour, respectively.

### 2.7. Ingredients for Biscuit Production

The biscuits were produced using the ingredients shown in Table 1 below.

### 2.8. Production of Biscuit

The biscuits were manufactured using the method published by Ogo et al. [12] with some adjustments. The already-weighed amounts of flour were mixed with the baking lard, sugar, and salt for 5 min to make a creamy dough. Except for the water, all of the remaining ingredients were added and mixed. The desired texture was achieved by gradually adding the measured amount of water while continuously mixing. Five minutes were spent kneading the dough on a clean, flat surface. After that, the dough was manually rolled into sheets and stamped into the desired shapes. The dough was placed in greased pans with fluid fat and baked for 18 min at 180 °C in a baking oven (Carma, Model 1945XL, Terim Group, Modena, Italy). The baked biscuits were then cooled and packaged

### 2.9. Methods of Analysis

#### 2.9.1. Nutritional Analysis of the Flours, Blends, and Biscuits

The proximate content of the biscuit was determined following the method outlined in the Association of Official Analytical Chemists Official Methods of Analysis of the Association of Official Analytical Chemists (AOAC) [13], with moisture content estimated separately. The protein content was assessed using the Kjeldahl method, while the fat content was determined using the Soxhlet method. Crude fibre and ash content in the product were also estimated. The carbohydrate content in the biscuit was obtained by subtracting the sum of moisture, protein, fat, fibre, and ash content per 100 g of the sample from 100.

#### 2.9.2. Proximate Analysis of Flours, Blends and Biscuits

Determination of moisture content: Moisture content was determined by the method described by [13]. Clean crucible dishes were dried for 1 h at 100 °C in a hot air oven to achieve constant weight, then cooled in a desiccator and weighed (w_1_). Two grams of each sample were placed in the various crucibles and dried at 100 °C until a consistent weight was achieved (w_2_). The dishes as well as the samples were put in a desiccator and weighed (w_3_).

(1) $$\%\;Moisture=\frac{W_{2}-W_{3}}{W_{2}-W_{1}}\times 100$$

where w_1_ = Weight of dish; w_2_ = Weight of dish + sample before drying; and w_3_ = Weight of dish + sample after drying.

Determination of crude protein content: The Kjeldahl method was used to determine crude protein [13]. In the Kjeldahl flask, two grams of sample were inserted. The flask was filled with anhydrous sodium sulphate (5 g Kjeldahl catalyst). With a few boiling chips, concentrated H_2_SO_4_ (25 mL) was added. In the fume chamber, the flask was heated until the sample solution became transparent. Before being transferred to a 250 mL volumetric flask, the sample solution was allowed to cool to room temperature, and distilled water was used to make it up to volume. The equipment was set up, and the distillation unit was cleaned. A distillate collector was filled with five millilitres of 2% boric acid solution and a few drops of methyl red indicator (100 mL conical flask). Under the condenser, a conical flask was inserted. The sample digest was then pipetted into the device in 5 mL and rinsed down with distilled water. The digest was given five millilitres of a 60 per cent sodium hydroxide (NaOH) solution. The sample was subjected to heat until it produced 100mL of distillate in the receiving flask. The receiving flask’s contents were titrated with 0.01 NHCl to a pink-coloured end point.

(2) $$\frac{T\times 14.01\times 0.01\times dilution\;factor}{2.0\times 1000}\times 100$$

where

T = titre value;
2.0 g = weight of the sample;
0.01 = molarity of HCl;
14.01 = atomic mass of nitrogen.
% Protein = % N × 6.25 (where 6.25 = conversion factor of protein).

Determination of fat: The fat content was determined using the Soxhlet extraction method according to [13]. A round bottom flask with a capacity of 500 mL was filled with 300 mL petroleum ether and attached to the Soxhlet extractor. In a designated thimble, two grams of the sample were inserted. Cotton wool was used to seal the extractor thimble. The equipment was heated for six hours and brought to a boil at 65 °C. The thimble was carefully removed. The petroleum ether was regained to be used again. The flask was removed and dried in an oven at 105 °C for 1 h once it was clear of ether (Carma, Model 1945XL, Terim Group Italy). The flask was weighed after cooling in a desiccator.

(3) $$\frac{Weight\;of\;Fat}{Weight\;of\;sample}\times 100$$

Determination of crude fibre: The crude fibre was determined using [13]. Three grams of the sample was weighed into a 50 mL beaker. The fat was extracted three times with petroleum ether, stirring, settling, and decanting each time. The material was air-dried before being transferred to a 600 mL dried beaker. The beaker was then filled with 200 mL of 1.25 per cent sulphuric acid and a few drops of anti-foaming agent. The beaker was subjected to heat for 30 min on digesting equipment with a pre-adjusted hot plate, swirling the beaker frequently to avoid solid clinging to the sides. The mixture was set aside for one minute after 30 min before being filtered through a Buchner funnel. The insoluble material was rinsed with boiling water until it was free of acid but did not break the suction. A wash bottle containing 200 mL of 1.25% sodium hydroxide solution was used to wash the residue back into the original flask. It was subjected to heat quickly for another 30 min, with the same care as previously. It was allowed to cool for one minute after 30 min of boiling before being filtered under suction. The residue was washed with 1% hydrochloric acid and boiling water. Boiling water was used to rinse the residue until it was acid-free. It was ether-rinsed three times and then alcohol-rinsed twice. The residue was placed in an ash plate and dried to a consistent weight at 100 °C. The ash was incinerated for 30 min at 600 °C, cooled in a desiccator, and weighed. The weight difference between the oven dry weight and the weight after incineration was used to calculate the sample’s fibre content. This was calculated as a percentage of the original sample weight used in the analysis.

(4) $$\frac{Weight\;after\;oven\;drying-weight\;after\;incineration}{Total\;weight\;of\;sample}\times 100$$

Determination of total ash: The AOAC [13] procedure was used to determine the amount of ash present. Two grams of sample were placed in a silica dish that had been ignited, cooled, and weighed. The dish and sample were lit slowly at first, then in a muffle furnace at 550 °C for 3 h, until a white or grey ash was formed. After cooling in a desiccator, the dish and its contents were weighed, and ash was obtained. The dish and contents were cooled in a desiccator and weighed.

(5) $$\%\;Ash=\frac{Weight\;of\;ash}{Weight\;of\;sample}\times 100$$

#### 2.9.3. Determination of Carbohydrate Content

The carbohydrate content of samples was evaluated by difference using the standard method of [13].

(6) % Carbohydrate = 100 − (% moisture + % fat + % ash + % protein + % crude fibre)

### 2.10. Storage Studies

The biscuits were stored on the laboratory shelf after packaging in a low-density polyethene bag and a carton inside polyethene, and evaluation was carried out for the following quality parameters: moisture content, water activity, microbial count, and crispiness. Over the two months of storage, samples were withdrawn at two)-week intervals. Sensory evaluation was used to subjectively determine the crispiness of the biscuits.

#### 2.10.1. Microbiological Analysis for Total Viable and Mould Counts

##### Total Viable Count

The pour plate method as described by [14] was adopted. One gram of the sample was macerated into nine millilitres of Ringer solution and well mixed by shaking. Then, 10^−2^ and 10^−3^ concentrations were obtained by diluting this. Then, by rocking the plates, 15 mL of sterile nutritional agar medium was poured and thoroughly mixed with the inoculum, and 0.1 mL dilution from each dilution bottle was poured into the corresponding plate. After the plates were incubated at 38 °C for 24 h, the colonies were counted and expressed as colony-forming units per gram (cfu/g).

(7) $$Original\;cell\;population\;\left(\frac{Cfu}{mL}\right)=\frac{Mean\;colony\;count\;per\;drop}{Estimated\;volume\;per\;drop\times Df}$$

##### Mould Count

The pour plate method as described by [14] was also used. Each dilution was put into corresponding plates with 0.1 mL of sample dilution, and 15 mL of sterile Sabouraud Dextrose Agar (SDA) medium was poured and thoroughly mixed with the inoculum by rocking the plates. Colonies formed on the plates were counted and expressed as colony-forming units per gram (cfu/g) after three days of incubation at room temperature.

Sensory evaluation: Sensory evaluation was conducted by 20 semi-trained panellists selected from students of the Department of Food Science and Technology, University of Nigeria, Nsukka using a nine-point Hedonic scale (1 = dislike extremely, 9 = like extremely), as defined by [15]. The appearance, consistency, flavour, taste, texture/crispiness, and general acceptance of the biscuits were all evaluated. Each biscuit sample was given a code and displayed on a plastic tray before a bright light. To prevent prejudice, the panellists were positioned properly. To mitigate any lingering effect between samples, the panellists were given water to drink during the test.

### 2.11. Data Analysis and Experimental Design

This study followed a Completely Randomized Design (CRD), and the data underwent one-way analysis of variance (ANOVA) using Statistical Packages for Service Solution (SPSS) version 23.0. To compare treatment means, Duncan’s new multiple range test (DNMRT) was employed, with statistical significance considered when *p* > 0.05 [16].

### 2.12. Pictorial Representation of Biscuits from Wheat and Okara Flour Blends

**Figure 1:**
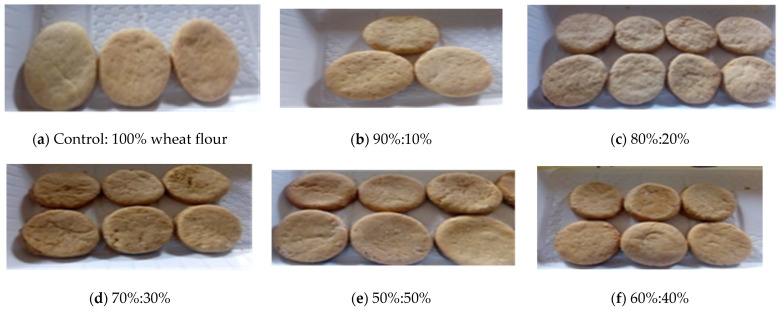
Biscuits made from wheat and okara flour blend ratios. Pictorial representation of biscuits formulated using varying proportions of wheat and okara flour (control: 100% wheat flour (**a**); 90:10 to 50:50 wheat/okara ratios (**b**–**f**).

Figure 1 shows the biscuits from wheat and okara flour blends using varying proportions of wheat and okara flour. The control group consists of biscuits made with 100% wheat flour, while the other biscuits are formulated with different wheat/okara ratios, ranging from 90:10 to 50:50.

### 2.13. Pictorial Representation of Biscuit from Wheat/Okara and Oyster Mushroom Flour

**Figure 2:**
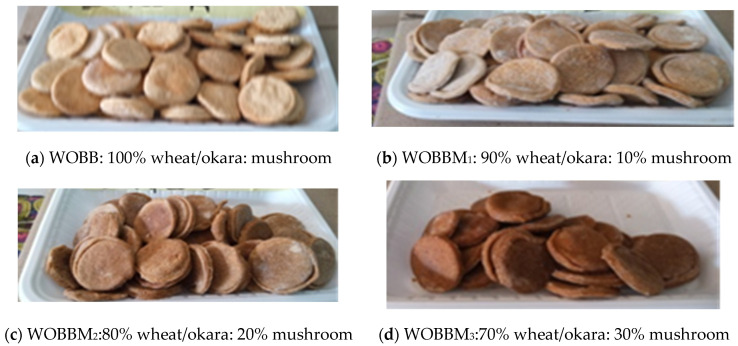

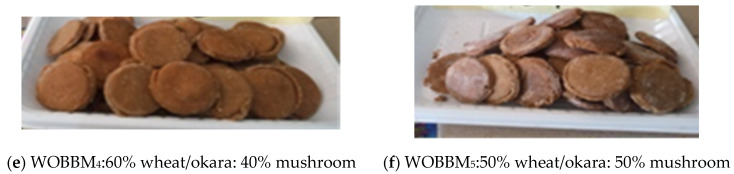
Biscuits made from wheat/okara and mushroom flour. Pictorial representation of biscuits formulated using wheat/okara composite flour and varying levels of mushroom flour (0–50%). WOBB: 100% wheat/okara composite flour; WOBBM1–WOBBM5: blends containing 10–50% mushroom flour.

Figure 2 shows biscuits formulated using wheat/okara composite flour and varying levels of mushroom flour (ranging from 0% to 50%).

## 3. Results

### 3.1. Proximate Composition of Unblended Flour from Wheat, Okara, and Mushroom

The analysis of the proximate composition of unblended flours from wheat, okara, and mushroom, as presented in Table 2, reveals significant differences in their nutritional profiles, as evidenced by varying levels of moisture, ash, protein, fibre, fat, and carbohydrate contents.

### 3.2. Moisture Content

The moisture content was measured as 6.12% for wheat flour, 6.68% for okara flour, and 7.22% for mushroom flour. Mushroom flour exhibited the highest moisture content, suggesting that its storage in moisture-proof containers could enhance shelf-life stability due to the relatively low moisture levels conducive to extended storage [17].

### 3.3. Ash Content

A significant variance was observed in the ash content, with mushroom flour registering the highest (7.56%), followed by okara flour (3.73%), and wheat flour showing the lowest (0.69%). These differences are notable as ash content is an indicator of the presence of nutritionally important minerals and substances in food [17].

### 3.4. Protein Content

The protein content was found to be considerably diverse among the flours, with wheat flour containing 12.63%, okara flour 30.39%, and mushroom flour 28.27%. This finding aligns with Enwere’s [18], who noted the substantial protein content in mushrooms. Unlike wheat, the proteins in okara and mushroom flour do not contribute to gluten formation, making them suitable for high-quality protein fortification in dietary biscuits [19].

### 3.5. Crude Fiber Content

The crude fibre content was highest in mushroom flour (9.49%), followed by okara flour (6.60%), and lowest in wheat flour (1.11%). High fibre content is beneficial in reducing blood cholesterol levels and moderating glucose absorption, thus aiding in blood glucose control and promoting digestive health [20].

### 3.6. Fat Content

A significant difference was observed in fat content across the flours. Okara flour contained the highest fat content at 10.83%, attributed to the high oil content in soybean germ [16]. Mushroom flour had a fat content of 5.95%, and wheat flour had the lowest at 1.01%. Notably, mushrooms are almost fat-free and devoid of cholesterol and trans-fats.

### 3.7. Carbohydrate Content

The carbohydrate content varied, with wheat flour having the highest (78.45%), while okara and mushroom flours had similar and lower carbohydrate levels (41.78% and 41.53%, respectively). This lower carbohydrate content reflects the intrinsic composition of these flours, which was minimally altered by processing.

In summary, the proximate analysis underscores the distinctive nutritional profiles of wheat, okara, and mushroom flours, each contributing unique qualities that can enhance the nutritional value of food products where they are used.

### 3.8. Proximate Composition (%) of the Blends of Wheat/Okara and Mushroom Flour

Our investigation, as detailed in Table 3, provides insightful data on the proximate composition of flour blends made from wheat, okara, and oyster mushrooms. The analysis revealed a moisture content range of 6.68% to 9.68% across the samples. Notably, the incorporation of mushroom flour resulted in a proportional increase in moisture content, with the blend containing 50% mushroom flour exhibiting the highest moisture levels. This observation aligns with the inherent moisture content of mushroom flour, recorded at 7.22%, and underscores the role of ingredient composition in determining the moisture profile of flour blends [11]. The ash content in the flour samples varied from 1.27% to 4.15%, indicating a significant rise upon the addition of mushroom flour. This trend is likely attributable to the higher ash content inherent in mushrooms, reflecting the presence of essential minerals in the flour blends.

Crucial to the nutritional value of the blends, the crude protein levels were found to range between 13.97% and 21.10%. This enhancement in protein content is particularly important given the relatively low protein and lysine levels in wheat flour, as noted by Agu [21]. In contrast, mushrooms are known for their high lysine content and overall protein richness, with a crude protein content of approximately 36% alongside a balanced profile of essential amino acids. This study also observed a variation in the total fat content of the flour blends, ranging from 2.63% to 5.81%, with an increase correlated to the proportion of mushroom flour added. This increment can be attributed to the distinct fat composition of mushrooms compared to wheat and okara.

A significant finding was the increase in fibre content, which ranged from 1.33% to 5.28% across the samples. The addition of mushroom flour notably elevated the fibre content, a critical component for dietary health. Lastly, the carbohydrate content of the blends showed a significant decrease (*p* < 0.05) with the addition of mushroom flour, ranging from 53.98% to 74.12%. This reduction is consistent with the lower carbohydrate levels typically found in mushrooms, as detailed in Table 2.

### 3.9. Proximate Content (%) of Biscuits Made from Wheat/Okara and Mushroom Flour Blends

Table 4 presents the proximate composition of biscuits formulated from varying blends of wheat/okara and mushroom flour. The analysis reveals distinct trends in moisture, ash, protein, fibre, fat, and carbohydrate content, correlating with the proportion of mushroom flour in the blends.

#### 3.9.1. Moisture Content

The moisture levels in the biscuits ranged from 4.19% to 4.89%. Biscuits with a 50% wheat/okara and 50% mushroom flour blend exhibited the highest moisture content, while those made entirely from wheat/okara flour (control) had the lowest. This increase in moisture with higher mushroom content can be attributed to the hygroscopic nature of the mushroom’s protein and fibre components, which are known to absorb and retain moisture during the baking process [22,23]. Lower moisture content in confectionaries is generally advantageous, reducing microbial growth and extending shelf life under appropriate storage conditions.

#### 3.9.2. Ash Content

The ash content varied from 1.19% to 4.59%, with the highest levels observed in the 50% wheat/okara and 50% mushroom flour blend. This increment in ash content with the addition of mushroom flour is significant (*p* < 0.05), likely due to the higher mineral content in mushrooms compared to wheat and okara.

#### 3.9.3. Protein Content

Protein levels in the biscuits spanned from 8.26% to 16.12%. Notably, biscuits with a 10% mushroom powder inclusion demonstrated a protein content of 10.07%, significantly higher than the control (8.26%). The highest protein content was found in biscuits with equal parts wheat/okara and mushroom flour, reflecting the high-quality protein profile of mushrooms (20–40% dry weight). This finding aligns with the known nutritional attributes of mushrooms as rich sources of protein [24,25].

#### 3.9.4. Fiber Content

The fibre content increased with the addition of mushroom flour, peaking in the 50% mushroom blend. This high fibre content is attributable to the dietary fibre richness of the mushroom flour used in the biscuit formulation, enhancing the overall fibre content and offering health benefits like improved digestion, commonly associated with high-fibre diets [25].

#### 3.9.5. Fat and Carbohydrate Content

Fat levels ranged from 14.28% to 16.19%, influenced by the higher fat content in okara and the oil absorption properties of mushrooms. Despite mushrooms being low in fat, the overall fat content in the biscuits is affected by other ingredients like margarine [26]. Carbohydrate content varied from 54.68% to 70.95%, with the lowest levels observed in the 50% wheat/okara and mushroom blend, suggesting that mushrooms contribute less to the carbohydrate profile compared to wheat.

These results indicate that incorporating mushroom flour into wheat/okara biscuits significantly enhances their nutritional profile, particularly in terms of protein and fibre content, while also impacting moisture, ash, fat, and carbohydrate levels.

### 3.10. Impact of Packaging Materials on Biscuit Quality During Storage

This study involved storing formulated biscuits at ambient temperature for two months, utilizing two types of packaging materials: low-density polyethylene (LDPE) and cartons. Assessments of moisture content, water activity, crispness, and microbial load were conducted bi-weekly. As Dixon [27] notes, the primary function of food packaging is to preserve and protect products from external factors that could compromise their quality, such as moisture, oxygen, light, and contamination.

### 3.11. Moisture Content Variations over Time

According to Table 5, the moisture content in the packaged biscuits varied between 4.19% and 6.85% during the storage period. The initial moisture content at week zero was the lowest, increasing to its peak at week four, followed by a decrease in weeks six and eight. This variation was statistically significant at a 5% probability level, as depicted in Table 5.

The observed increase in moisture content during the initial weeks can be attributed to water vapour migration from the storage environment into the packaging material and condensation within the package. This phenomenon was more effectively mitigated by the carton within the polyethylene bag than by LDPE bags alone. The dry, hygroscopic nature of the biscuits, coupled with environmental factors like temperature and relative humidity, and the properties of the packaging materials, significantly influenced this moisture gain, in line with the findings of Nagi et al. [28]. These results underscore the importance of choosing appropriate packaging materials to maintain the quality of food products during storage. The interaction between the packaging, the ambient environment, and the product’s characteristics plays a crucial role in preserving the desired moisture level and, by extension, the overall quality and shelf-life of the biscuits.

### 3.12. Water Activity

Table 6 shows the impact of the storage and packaging material on the water activity of the biscuits stored under ambient conditions, with sample WOBB having the lowest values and WOBBM_5_ samples having the highest values ranging from 0.32 to 0.55. All samples were significantly (*p* < 0.05) different from the others. The results demonstrated a similar response to the moisture content of the water activity of the stored product. Water activity is therefore directly related to humidity. The water activity was found to increase within the first to fourth weeks of storage and decreased in the sixth and eighth weeks in both packaging materials. The carton-inside-polyethylene packaging material (CPP) is found to have better protection concerning water activity. Water activity is a major component of microbial growth and a crucial component affecting all bacteria. Dry foods normally contain less than 10 per cent moisture and 0.6 or below water activity.

### 3.13. Total Viable and Mould Count of Biscuits During Storage

The effects of storage and packaging material on the total viable count and mould count of the biscuits are shown in Table 7 and Table 8. The values obtained from the formulated biscuits for mould count ranged from 0.0 × 10 to 6.0 × 10 cfu/g in the PP packaging, with sample WOBBM_5_ having the highest value (6.0 × 10 cfu/g) at week 8 and sample WOBB having the least value (0.0 × 10 cfu/g) at week 0. The values obtained for the LDPE packaging material for mould ranged from 0.0 × 10 to 6.0 × 10 cfu/g. Sample WOBB had the least obtained value at week 0, while WOBBM_5_ had the highest value at week 8. The total viable count of the biscuits for the PP packaging ranged from 3.1 × 10 to 6.8 × 10^4^ cfu/g, with sample WOBB (control) having the lowest value and sample WOBBM_5_ (50:50) having the highest value. The mould growth in the samples could be attributed to the moisture content of the formulated samples as well the higher amount of oyster mushroom in those samples and its ability to absorb and hold water. A significant (*p* > 0.05) increase in microbial load was observed in both packaging materials (0.0 × 10 to 6 × 10 cfu/g) for mould as the storage weeks increased, and the biscuits had slight whitish discolouration. The trend observed indicates that the mould growth in biscuits stored at ambient temperature was significantly influenced by the a_w_ level and moisture content of the biscuits. The mould-forming unit for a colony was dramatically increased at week 2 of storage from 0.0 to 1.10 cfu/g, with the degree of water activity increasing from 0.32 ± 0.00 to 0.35 ± 0.00. In terms of mould growth, storage in the CPP packaging material was a better barrier. The temperature and packaging material had substantial effects on the microbial load. For the LDPE packaging material under ambient conditions, after a sixth week of storage, visual observations of mould growth were initially detected. The effects of high relative humidity, which contributed to the rise of water activities and the percentage of moisture content in biscuits, could be linked to the deterioration seen. As a result, this condition increased microbe proliferation. When analysed according to the Microbiological Quality Guidelines, 10^4^ cfu/g was found to be satisfactory, 10^4^ to 10^5^ cfu/g acceptable, and 10^6^ cfu/g and above was of unsatisfactory quality; the total viable and mould count of the samples was not high.

The evaluation of crispness, a critical quality parameter in biscuit production, was conducted to understand how storage and packaging affect the textural attributes of biscuits formulated from wheat, okara, and mushroom blends. Crispness degradation, alongside lipid oxidation, is pivotal in determining the quality of biscuits during storage, particularly concerning moisture absorption and fat content, which often exceeds 20%, and the resultant aroma loss [29].

### 3.14. Crispness Evaluation over Storage Period

Table 9 delineates the variation in crispness of the biscuits over an eight-week storage period under different packaging conditions. The measurement of crispness was quantified using a scale where lower values indicate less crispness. A positive and significant correlation was exhibited in mushroom/okara flour with greater water retention than wheat flour.

Polypropylene (PP) Bulk Packaging: In this packaging format, crispness values ranged from 0.30 in the WOBB sample (week 6) to 0.50 in the WOBBM_5_ sample (week 8). Notably, every sample exhibited significant changes (*p* < 0.05) in crispness throughout the storage period. The WOBBM_5_ sample showed the lowest crispness at week 4, while the WOBB sample had the highest at week 6.

Low-Density Polyethylene (LDPE) Packaging: The crispness scores in LDPE packaging varied from 4.20 to 7.70. Similar to PP packaging, the WOBBM_5_ sample demonstrated the lowest crispness at week 4, and the WOBB sample exhibited the highest crispness at week 6. All samples presented significant differences (*p* < 0.05) in crispness.

### 3.15. Trends in Crispness over Time

The analysis revealed a trend where crispness initially increased during the first two weeks of storage, followed by a decline in the fourth week and then a subsequent increase in the sixth and eighth weeks. This trend was consistent across both packaging materials, suggesting that the crispness of the biscuits is influenced by storage duration and conditions.

Our study contributes to the field of Food Science by exploring alternative, sustainable sources of flour in biscuit production. The integration of oyster mushroom (*Pleurotus ostreatus*) and okara flour represents a novel approach in the context of Nigerian agricultural practices and food production. To ensure replicability and consistent characterization, the raw materials—wheat flour, soybean seeds for okara, and oyster mushrooms—were sourced directly from consistent producers over two harvest years. This approach aligns with the standards for novel food characterization, ensuring that our findings are replicable and relevant to other researchers and practitioners in the field [30]. We focused on the nutritional composition by quantifying the levels of bioactive compounds, particularly focusing on protein and dietary fibre content, which are crucial for addressing malnutrition [31]. The increase in protein content from 8.26% to 16.12% and the corresponding increase in fibre demonstrate the practicality of incorporating these bioactive compounds at levels that are significant yet achievable in a standard biscuit production process. This aligns with the need for practicality in the incorporation of bioactive compounds as emphasized by [32] in the context of food science research. The use of a common food product like biscuits allows for a realistic assessment of how these bioactive compounds might be absorbed and utilized in the human body, which is a critical aspect of food science research, especially when introducing new ingredients into traditional food items [33]. Future iterations of this research will aim to balance these aspects to ensure that the final product is not only nutritionally superior but also appealing to consumers.

## 4. Future Research

One of the key limitations of this study is the absence of bioavailability testing, which is essential for understanding how the increased protein and fibre content from okara flour is absorbed and utilized by the human body. Although the study result (the biscuit model) could serve as a practical food matrix for bioavailability testing, this was not included in the present study. Additionally, product quality was primarily assessed through basic proximate testing, with no evaluation of other important factors such as sensory characteristics, texture, and shelf life, which could affect consumer acceptance and product viability.

To address these limitations, future research should prioritize bioavailability testing to provide a deeper understanding of the nutritional benefits of the formulated biscuits. Further investigation into consumer preferences and scalability for commercial production would also be valuable in assessing the market potential. Additionally, future studies should expand product quality evaluations to include sensory attributes and other relevant factors, offering a more comprehensive analysis of the biscuits’ viability in the food industry.

Future research from this study can investigate the maximum level of mushroom flour enrichment in wheat/okara flour for biscuit production at 20% since higher levels reduce the product’s consumer appeal. Further research, particularly through bioassays, is needed to understand the effects of these biscuits on health and nutrition more comprehensively. While this study provides a foundation for understanding the economic feasibility, future research will include a comprehensive cost–benefit analysis. This will compare the full production costs, including raw materials and processing, with the market pricing of enriched biscuits versus traditional wheat-based biscuits. It will also evaluate consumer acceptance and willingness to pay for these products.

## 5. Conclusions

This study demonstrates a promising approach to enhancing the nutritional value of biscuits using oyster mushrooms and okara flour. However, it is also recognized that any alterations to traditional food products must consider consumer preferences, especially in terms of taste and texture. After 2 months of storage, biscuits stored in cartons inside polyethene were more shelf-stable than those in LDPE bags, highlighting the importance of packaging in the shelf life of these novel food products. The inclusion of mushrooms in biscuits, therefore, results in high-quality products that can contribute to addressing global malnutrition challenges.

## Figures and Tables

**Table 1:** Ingredients for biscuit production.

Ingredients	Quantity
Flour blends	100 g
Salt (table)	0.3 g
Baking milk	5 g
Baking powder	1 g
Beaten egg	10 g
Sugar (granulated)	20 g
Baking fat	22 g
Water	45 mL
Flavour (vanilla)	10 mL

Source: [4].

**Table 2:** Proximate composition (%) of unblended flour from wheat, mushroom, and okara.

Sample	Moisture (%)	Ash (%)	Protein (%)	Fiber (%)	Fat (%)	Carbohydrate (%)
WHF	6.12 ^a^ ± 0.16	0.69 ^a^ ± 0.22	12.63 ^a^ ± 0.01	1.11 ^a^ ± 0.71	1.01 ^a^ ± 0.27	78.45 ^b^ ± 0.39
MRF	7.22 ^c^ ± 0.09	7.56 ^c^ ± 0.06	28.27 ^b^ ± 0.10	9.49 ^c^ ± 0.24	5.95 ^b^ ± 0.13	41.53 ^a^ ± 0.03
OKF	6.68 ^b^ ± 0.21	3.73 ^b^ ± 0.11	30.39 ^b^ ± 0.04	6.60 ^b^ ± 0.18	10.83 ^c^ ± 0.24	41.78 ^a^ ± 0.57

Values are means ± SD of duplicate determinations. Value in the same column with different superscripts were significantly (*p* < 0.05) different. Key: WHF = 100% wheat flour; MRF = 100% mushroom flour; OKF = 100% okara flour.

**Table 3:** Proximate composition of the blends of wheat/okara and mushroom flour.

Samples	Moisture (%)	Ash (%)	Protein (%)	Fiber (%)	Fat (%)	Carbohydrate (%)
WOBBF	6.68 ^a^ ± 0.23	1.27 ^a^ ± 0.04	13.97 ^a^ ± 0.12	1.33 ^a^ ± 0.12	2.63 ^a^ ± 0.52	74.12 ^f^ ± 0.56
WOBBMF_1_	7.20 ^b^ ± 0.17	2.75 ^b^ ± 0.14	15.90 ^b^ ± 0.15	2.30 ^b^ ± 0.14	4.35 ^b^ ± 0.28	67.50 ^e^ ± 0.63
WOBBMF_2_	7.74 ^c^ ± 0.30	3.72 ^c^ ± 0.08	16.97 ^c^ ± 0.63	3.00 ^c^ ± 0.28	5.68 ^c^ ± 0.24	62.89 ^d^ ± 0.56
WOBBMF_3_	8.82 ^d^ ± 0.12	3.68 ^d^ ± 0.42	18.91 ^d^ ± 0.41	3.95 ^d^ ± 0.13	5.74 ^c^ ± 0.18	58.90 ^c^ ± 0.61
WOBBMF_4_	8.86 ^e^ ± 0.18	3.79 ^d^ ± 0.01	20.06 ^e^ ± 0.33	4.62 ^e^ ± 0.16	5.80 ^c^ ± 0.13	56.87 ^b^ ± 0.16
WOBBMF_5_	9.68 ^f^ ± 0.21	4.15 ^e^ ± 0.03	21.10 ^f^ ± 0.14	5.28 ^f^ ± 0.39	5.81 ^c^ ± 0.09	53.98 ^a^ ± 0.69

Values are means ± SD of duplicate determinations. Value in the same column with different superscripts was significantly (*p* < 0.05) different. Key: WOBBF = 100% wheat/okara; WOBBMF_1_ = 90%wheat/okara and 10% mushroom flour; WOBBMF_2_ = 80% wheat/okara and 20% mushroom flour; WOBBMF_3_ = 70% wheat/okara and 30% mushroom flour; WOBBMF_4_ = 60% wheat/okara and 40% mushroom flour; WOBBMF_5_ = 50% wheat/okara and mushroom flour.

**Table 4:** Proximate composition of biscuits made from wheat/okara and mushroom flour.

Samples	Moisture (%)	Ash (%)	Protein (%)	Fibre (%)	Fat (%)	Carbohydrate (%)
WOBB	4.19 ^a^ ± 0.39	1.19 ^a^ ± 0.02	8.26 ^a^ ± 0.07	1.13 ^a^ ± 0.28	14.28 ^a^ ± 0.58	70.95 ^f^ ± 0.91
WOBBM_1_	4.71 ^b^ ± 0.10	1.87 ^b^ ± 0.04	10.07 ^b^ ± 0.14	1.72 ^b^ ± 0.35	15.34 ^ab^ ± 0.55	66.29 ^e^ ± 0.43
WOBBM_2_	4.74 ^b^ ± 0.19	2.20 ^c^ ± 0.04	12.52 ^c^ ± 0.28	2.45 ^c^ ± 0.26	15.38 ^ab^ ± 0.59	62.71 ^d^ ± 0.76
WOBBM_3_	4.82 ^c^ ± 0.06	3.33 ^d^ ± 0.10	13.03 ^c^ ± 0.50	2.76 ^cd^ ± 0.09	15.41 ^ab^ ± 0.58	60.65 ^c^ ± 0.15
WOBBM_4_	4.86 ^c^ ± 0.02	4.12 ^e^ ± 0.16	15.25 ^d^ ± 0.14	3.06 ^de^ ± 0.13	16.10 ^b^ ± 0.28	56.61 ^b^ ± 0.21
WOBBM_5_	4.89 ^cd^ ± 0.05	4.59 ^f^ ± 0.77	16.12 ^e^ ± 0.38	3.53 ^e^ ± 0.38	16.19 ^b^ ± 0.30	54.68 ^a^ ± 0.10

Proximate composition (%) of biscuits formulated from varying ratios of wheat/okara and mushroom flour. Values are means ± SD of duplicate determinations. Values in the same column with different superscripts were significantly (*p* < 0.05) different. Key: WOBB = 100% wheat/okara; WOBBM_1_ = 90% wheat/okara and 10% mushroom flour; WOBBM_2_ = 80% wheat/okara and 20% mushroom flour; WOBBM_3_ = 70% wheat/okara and 30% mushroom flour; WOBBM_4_ = 60% wheat/okara and 40% mushroom flour; WOBBM_5_ = 50% wheat/okara and mushroom flour.

**Table 5:** Effect of storage and packaging material (PP and LDPE) on moisture content (%) in biscuits over 8 weeks.

Samples	Packaging Material	0	Storage 2	Periods 4	(Weeks) 6	8
WOBB	PP	4.19 ^b^ ± 0.12	5.67 ^d^ ± 0.34	5.61 ^c^ ± 0.20	4.11 ^a^ ± 0.10	4.10 ^a^ ± 0.22
	LDPE	4.19 ^b^ ± 0.12	5.72 ^d^ ± 0.11	5.40 ^c^ ± 0.08	4.20 ^b^ ± 0.04	4.11 ^a^ ± 0.10
WOBBM_1_	PP	4.71 ^c^ ± 0.04	5.77 ^e^ ± 0.14	5.42 ^d^ ± 0.10	4.32 ^a^ ± 0.08	4.50 ^b^ ± 0.08
	LDPE	4.71 ^c^ ± 0.04	5.96 ^e^ ± 0.19	5.66 ^d^ ± 0.22	4.40 ^b^ ± 0.03	4.20 ^a^ ± 0.11
WOBBM_2_	PP	4.74 ^b^ ± 0.03	6.01 ^d^ ± 0.11	5.61 ^c^ ± 0.19	4.35 ^a^ ± 0.03	4.73 ^b^ ± 0.04
	LDPE	4.74 ^c^ ± 0.03	6.14 ^e^ ± 0.08	5.70 ^d^ ± 0.08	4.45 ^a^ ± 0.03	4.53 ^b^ ± 0.03
WOBBM_3_	PP	4.82 ^b^ ± 0.01	5.89 ^e^ ± 0.03	5.54 ^d^ ± 0.04	4.62 ^a^ ± 0.04	5.00 ^c^ ± 0.00
	LDPE	4.82 ^b^ ± 0.01	6.20 ^e^ ± 0.04	5.80 ^d^ ± 0.11	4.70 ^a^ ± 0.05	5.20 ^c^ ± 0.02
WOBBM_4_	PP	4.86 ^b^ ± 0.03	6.11 ^d^ ± 0.06	6.73 ^e^ ± 0.02	4.70 ^a^ ± 0.02	5.50 ^c^ ± 0.03
	LDPE	4.86 ^b^ ± 0.03	6.25 ^d^ ± 0.03	6.80 ^e^ ± 0.03	4.76 ^a^ ± 0.18	6.00 ^c^ ± 0.02
WOBBM_5_	PP	4.89 ^b^ ± 0.18	6.15 ^d^ ± 0.08	6.76 ^e^ ± 0.04	4.75 ^a^ ± 0.03	6.00 ^c^ ± 0.02
	LDPE	4.89 ^b^ ± 0.18	6.25 ^d^ ± 0.03	6.85 ^e^ ± 0.03	4.86 ^a^ ± 0.04	6.20 ^c^ ± 0.03

Values are means ± SD of duplicate determinations. Value in the same column with different superscripts was significantly (*p* ˂ 0.05) different. Key: WOBB = 100% wheat/okara; WOBBM_1_ = 90% wheat/okara and 10% mushroom flour; WOBBM_2_ = 80% wheat/okara and 20% mushroom flour; WOBBM_3_ = 70% wheat/okara and 30% mushroom flour; WOBBM_4_ = 60% wheat/okara and 40% mushroom flour; WOBBMF_5_ = 50% wheat/okara and mushroom flour; PP = paper inside polyethylene bag (bulk packaging); LDPE = low-density polyethylene bag.

**Table 6:** Effect of storage and packaging material on the water activity of the biscuits.

Samples	Packaging Material	0	Storage 2	Period 4	(Weeks) 6	8
WOBB	PP	0.32 ^b^ ± 0.00	0.35 ^c^ ± 0.00	0.34 ^c^ ± 0.00	0.30 ^a^ ± 0.02	0.41 ^d^ ± 0.03
	LDPE	0.32 ^a^ ± 0.00	0.35 ^b^ ± 0.00	0.34 ^ab^ ± 0.00	0.33 ^a^ ± 0.03	0.45 ^c^ ± 0.04
WOBBM_1_	PP	0.36 ^b^ ± 0.00	0.37 ^b^ ± 0.01	0.36 ^b^ ± 0.00	0.34 ^a^ ± 0.00	0.42 ^c^ ± 0.02
	LDPE	0.36 ^a^ ± 0.00	0.38 ^ab^ ± 0.02	0.37 ^a^ ± 0.01	0.36 ^a^ ± 0.00	0.45 ^b^ ± 0.03
WOBBM_2_	PP	0.40 ^c^ ± 0.00	0.40 ^c^ ± 0.00	0.40 ^b^ ± 0.02	0.36 ^a^ ± 0.00	0.45 ^d^ ± 0.03
	LDPE	0.40 ^b^ ± 0.00	0.41 ^b^ ± 0.03	0.41 ^b^ ± 0.00	0.37 ^a^ ± 0.01	0.46 ^c^ ± 0.02
WOBBM_3_	PP	0.45 ^b^ ± 0.00	0.46 ^b^ ± 0.02	0.45 ^b^ ± 0.00	0.40 ^a^ ± 0.00	0.50 ^c^ ± 0.00
	LDPE	0.45 ^b^ ± 0.00	0.48 ^c^ ± 0.02	0.45 ^b^ ± 0.00	0.40 ^a^ ± 0.00	0.46 ^b^ ± 0.02
WOBBM_4_	PP	0.45 ^b^ ± 0.00	0.48 ^c^ ± 0.01	0.45 ^b^ ± 0.02	0.41 ^a^ ± 0.00	0.50 ^d^ ± 0.03
WOBBM_5_	LDPE	0.45 ^b^ ± 0.00	0.48 ^c^ ± 0.01	0.46 ^b^ ± 0.01	0.41 ^a^ ± 0.00	0.47 ^c^ ± 0.04
	PP	0.46 ^b^ ± 0.00	0.48 ^c^ ± 0.01	0.46 ^b^ ± 0.01	0.41 ^a^ ± 0.00	0.50 ^d^ ± 0.02
	LDPE	0.46 ^b^ ± 0.00	0.50 ^c^ ± 0.00	0.47 ^b^ ± 0.01	0.41 ^a^ ± 0.00	0.55 ^c^ ± 0.00

Values are means ± SD of duplicate determinations. Value in the same column with different superscripts were significantly (*p* ˂ 0.05) different. Key: WOBB = 100% wheat/okara; WOBBM_1_ = 90% wheat/okara and 10% mushroom flour; WOBBM_2_ = 80% wheat/okara and 20% mushroom flour; WOBBM_3_ = 70% wheat/okara and 30% mushroom flour; WOBBM_4_ = 60% wheat/okara and 40% mushroom flour; WOBBMF_5_ = 50% wheat/okara and mushroom flour, PP = paper in polyethylene (bulk packaging); LDPE = low-density polyethylene.

**Table 7:** Effect of storage and packaging material (paper in polyethene/bulk packaging) on microbial counts (TVC and mould growth) in biscuits over 8 weeks.

Samples	Packaging Material	Microbial Count (cfu/g)	0	Storage 2	Period 4	(Weeks) 6	8
WOBB	PP	TVC	3.1 × 10	3.4 × 10	3.6 × 10^2^	4.8 × 10^2^	2.0 × 10^3^
		Mould	ND	1.0 × 10	1.0 × 10	2.0 × 10	3.0 × 10
WOBBM_1_	PP	TVC	4.8 × 10	3.8 × 10^2^	6.2 × 10^2^	7.0 × 10^2^	2.0 × 10^3^
		Mould	ND	1.0 × 10	2.0 × 10	2.0 × 10	3.0 × 10
WOBBM_2_	PP	TVC	6.3 × 10	4.2 × 10^2^	6.8 × 10^2^	8.3 × 10^3^	2.6 × 10^4^
		Mould	ND	1.0 × 10	2.0 × 10	4.0 × 10	4.0 × 10
WOBBM_3_	PP	TVC	9.2 × 10	4.4 × 10^2^	1.0 × 10^3^	1.2 × 10^4^	3.2 × 10^4^
		Mould	ND	1.0 × 10	2.0 × 10	4.0 × 10	4.0 × 10
WOBBM_4_	PP	TVC	1.0 × 10^2^	5.2 × 10^2^	1.3 × 10^3^	1.6 × 10^4^	4.5 × 10^4^
		Mould	ND	1.0 × 10	2.0 × 10	5.0 × 10	5.0 × 10
WOBBM_5_	PP	TVC	1.2 × 10^2^	5.8 × 10^2^	3.8 × 10^3^	1.8 × 10^4^	6.8 × 10^4^
		Mould	ND	2.0 × 10	4.0 × 10	5.0 × 10	6.0 × 10

Values are means ± SD of duplicate determinations. Key: WOBB = 100% wheat/okara; WOBBM_1_ = 90% wheat/okara and 10% mushroom flour; WOBBM_2_ = 80% wheat/okara and 20% mushroom flour; WOBBM_3_ = 70% wheat/okara and 30% mushroom flour; WOBBM_4_ = 60% wheat/okara and 40% mushroom flour; WOBBM_5_ = 50% wheat/okara and mushroom flour; PP = paper in polyethylene (bulk packaging); TVC = total viable count; ND = not detected.

**Table 8:** Effect of storage and packaging material (low-density polyethene) on the microbial counts of the biscuits.

Samples	Packaging Material	Microbial Count (cfu/g)	0	Storage 2	Period 4	(Weeks) 6	8
WOBB	LDPE	TVC	3.1 × 10	3.8 × 10^2^	4.0 × 10^2^	6.6 × 10^2^	2.0 × 10^3^
		Mould	ND	1.0 × 10	2.0 × 10	2.0 × 10	3.0 × 10
WOBBM_1_	LDPE	TVC	4.8 × 10	4.0 × 10^2^	8.0 × 10^2^	8.8 × 10^2^	2.3 × 10^3^
		Mould	ND	1.0 × 10	2.0 × 10	2.0 × 10	3.0 × 10
WOBBM_2_	LDPE	TVC	6.3 × 10	4.4 × 10^2^	9.3 × 10^2^	1.0 × 10^4^	3.3 × 10^4^
		Mould	ND	1.0 × 10	4.0 × 10	4.0 × 10	5.0 × 10
WOBBM_3_	LDPE	TVC	9.2 × 10	4.9 × 10^2^	1.2 × 10^3^	1.5 × 10^4^	4.7 × 10^4^
		Mould	ND	1.0 × 10	4.0 × 10	4.0 × 10	5.0 × 10
WOBBM_4_	LDPE	TVC	1.0 × 10^2^	5.8 × 10^2^	1.5 × 10^3^	2.3 × 10^4^	6.2 × 10^4^
		Mould	ND	2.0 × 10	5.0 × 10	5.0 × 10	6.0 × 10
WOBBM_5_	LDPE	TVC	1.2 × 10^2^	7.6 × 10^2^	4.8 × 10^3^	1.8 × 10^4^	7.2 × 10^4^
		Mould	ND	2.0 × 10	5.0 × 10	5.0 × 10	6.0 × 10

Values are means ± SD of duplicate determinations. Key: WOBB = 100% wheat/okara; WOBBM_1_ = 90% wheat/okara and 10% mushroom flour; WOBBM_2_ = 80% wheat/okara and 20% mushroom flour; WOBBM_3_ = 70% wheat/okara and 30% mushroom flour; WOBBM_4_ = 60% wheat/okara and 40% mushroom flour; WOBBMF_5_ = 50% wheat/okara and mushroom flour; LDPE = low-density polyethylene; TVC = total viable count; ND = not detected.

**Table 9:** Crispness evaluation of biscuits.

Samples	Packaging Material	0	Storage 2	Period 4	(Weeks) 6	8
WOBB	PP	7.10 ^b^ ± 0.01	7.20 ^c^ ± 0.01	6.50 ^a^ ± 0.30	7.80 ^e^ ± 0.00	7.60 ^d^ ± 0.24
	LDPE	7.10 ^d^ ± 0.01	7.00 ^c^ ± 0.01	6.30 ^b^ ± 0.12	7.70 ^e^ ± 0.30	6.00 ^a^ ± 0.00
WOBBM_1_	PP	6.90 ^c^ ± 0.40	6.80 ^b^ ± 0.00	6.40 ^a^ ± 0.20	7.20 ^e^ ± 0.03	7.00 ^d^ ± 0.04
	LDPE	6.90 ^d^ ± 0.10	6.80 ^c^ ± 0.00	6.30 ^b^ ± 0.30	7.20 ^e^ ± 0.03	5.80 ^a^ ± 0.06
WOBBM_2_	PP	5.30 ^b^ ± 0.01	5.50 ^c^ ± 0.00	5.00 ^a^ ± 0.00	6.40 ^d^ ± 0.00	6.80 ^e^ ± 1.16
	LDPE	5.30 ^b^ ± 0.01	5.50 ^c^ ± 0.00	4.80 ^a^ ± 0.06	6.30 ^d^ ± 0.02	5.50 ^c^ ± 0.30
WOBBM_3_	PP	5.40 ^b^ ± 0.01	5.80 ^c^ ± 0.00	4.70 ^a^ ± 0.03	5.80 ^c^ ± 0.08	6.50 ^d^ ± 0.20
	LDPE	5.40 ^c^ ± 0.01	5.80 ^d^ ± 0.00	4.60 ^a^ ± 0.04	5.90 ^e^ ± 0.03	5.00 ^b^ ± 0.00
WOBBM_4_	PP	4.60 ^c^ ± 0.01	4.60 ^c^ ± 0.00	4.30 ^a^ ± 001	5.60 ^e^ ± 0.20	5.00 ^d^ ± 0.00
WOBBM_5_	LDPE	4.60 ^d^ ± 0.01	4.60 ^d^ ± 0.00	4.20 ^a^ ± 0.03	5.30 ^e^ ± 0.03	4.50 ^c^ ± 0.30
	PP	4.60 ^a^ ± 0.06	4.60 ^a^ ± 0.00	4.60 ^a^ ± 0.04	5.00 ^b^ ± 0.00	5.30 ^c^ ± 0.03
	LDPE	4.60 ^c^ ± 0.06	4.60 ^c^ ± 0.00	4.20 ^a^ ± 0.03	5.10 ^d^ ± 0.02	4.30 ^b^ ± 0.03

Effect of storage period and packaging material (PP and LDPE) on the crispness of biscuits formulated with wheat/okara and mushroom flour. Values are means ± SD of duplicate determinations. Value in the same column with different superscripts were significantly (*p* ˂ 0.05) different. Key: WOBB = 100% wheat/okara; WOBBM_1_ = 90% wheat/okara and 10% mushroom flour; WOBBM_2_ = 80% wheat/okara and 20% mushroom flour; WOBBM_3_ = 70% wheat/okara and 30% mushroom flour; WOBBM_4_ = 60% wheat/okara and 40% mushroom flour; WOBBM_5_ = 50% wheat/okara and mushroom flour; PP = paper in polyethene (bulk packaging); LDPE = low-density polyethene (Ziploc).

## Data Availability

The data available in this manuscript are available upon request from the authors.
